# Programmable RNA therapeutics: converging vaccination and on-demand disease treatment via self-adaptive RNA platforms

**DOI:** 10.3389/fimmu.2026.1872123

**Published:** 2026-10-29

**Authors:** Okechukwu Paul-Chima Ugwu, Michael Ben Okon, Fabian Chukwudi Ogenyi, Jovita Nnenna Ugwu, Chinyere N. Ugwu

**Affiliations:** Department of Research, Publication and Extension, Kampala International University, Kampala, Uganda

**Keywords:** cancer vaccines, lipid nanoparticles, mRNA therapeutics, personalised medicine, RNA-based nanomedicine, self-amplifying RNA, synthetic biology, vaccines

## Abstract

RNA technology, most prominently messenger RNA (mRNA), has transformed vaccine development by enabling rapid, sequence-specific antigen design, scalable manufacturing, and transient protein expression. Its long-term significance, however, extends beyond prophylactic vaccination. This Perspective argues that mRNA should be conceptualised as a programmable therapeutic platform capable of integrating vaccination, immune modulation, and on-demand disease treatment. Current mRNA vaccines deliver predefined antigenic instructions, whereas emerging RNA systems, including self-amplifying RNA (saRNA), circular RNA, sequence-engineered mRNA, and targeted lipid nanoparticles (LNPs), may support more adaptable therapeutic functions. Such platforms could prime immunity, replace deficient proteins, encode antibodies, reprogramme immune cells, or induce antigen-specific tolerance. The concept of self-adaptive RNA-based therapeutics remains at an early stage and should not be overstated; most existing systems are programmable at the design stage rather than biologically autonomous after administration. Nonetheless, this conceptual position is no longer purely speculative: the first regulatory approval of a self-amplifying RNA vaccine and controlled-trial evidence of clinical benefit from a personalised neoantigen mRNA therapeutic in resected melanoma indicate that programmable RNA platforms are beginning to move from concept to clinical validation across both the prophylactic and therapeutic ends of the spectrum. Key challenges include tissue-specific delivery, expression control, innate immune activation, repeat dosing, safety assessment, regulatory classification, and equitable access. A self-adaptive RNA-based framework does not replace existing vaccines or biologics but extends them towards responsive, personalised, and temporally controlled therapy.

## Introduction

Messenger RNA has advanced from an experimental molecule to a clinically validated therapeutic platform. The rapid development of COVID-19 mRNA vaccines demonstrated that synthetic RNA can be designed, manufactured, and deployed at scale (1, 2), an achievement facilitated by advances in RNA modification, sequence optimisation, LNP delivery, and attenuation of innate immune sensing (3, 4). This clinical trajectory has continued: a self-amplifying mRNA vaccine has since received the first regulatory approval granted to this RNA class, and subsequent phase 3 trials have confirmed durable immunogenicity with this lower-dose platform (5, 6).

The vaccine paradigm, however, represents only one dimension of mRNA biology. Unlike DNA-based approaches, mRNA does not require nuclear entry and does not integrate into the host genome, enabling transient protein expression suited to prophylactic vaccination, protein replacement, cancer immunotherapy, antibody expression, immune-cell programming, and tolerogenic therapy. This Perspective proposes that the next stage of RNA-based nanomedicine will be characterised by convergence: programmable RNA platforms that unite vaccination and disease treatment within adaptable therapeutic architectures, a proposition increasingly supported by controlled clinical data rather than preclinical signal alone.

## From vaccination to therapeutic programming

Conventional mRNA vaccines instruct host cells to produce defined antigens that stimulate adaptive immunity, a strategy valued for its capacity to allow rapid antigen redesign when pathogens evolve (2). Many diseases, however, require more than immune priming. Cancer, autoimmune disease, fibrosis, and genetic disorders may each necessitate temporary expression of antibodies, cytokines, enzymes, receptors, or gene-editing components.

Early evidence supports this wider therapeutic scope. Personalised neoantigen vaccination has demonstrated feasibility and immunogenicity in melanoma (7, 8), and this feasibility signal has since matured into controlled evidence of clinical benefit: a randomised phase 2b trial of an individualised neoantigen mRNA therapeutic combined with pembrolizumab reduced recurrence risk by 44% compared with pembrolizumab alone in resected high-risk melanoma (9). In pancreatic cancer, a comparably personalised mRNA neoantigen vaccine generated detectable T-cell responses in half of treated patients, with immune responders showing longer recurrence-free survival than non-responders (10). mRNA-encoded bispecific antibodies eliminated large tumours in preclinical models (11), and modified mRNA delivered in LNPs generated transient chimeric antigen receptor (CAR) T cells *in vivo* in a cardiac injury model (12). Taken together, these findings confirm that mRNA can function not only as a vaccine template but also as a temporary therapeutic instruction set, and that this function is now supported by randomised clinical data rather than mechanistic plausibility alone.

## Self-adaptive RNA-based platforms

Self-adaptive RNA-based therapeutics are defined here as systems designed to adjust therapeutic output according to disease context, tissue environment, or intended immune state. In the near term, adaptivity is likely to be modular rather than fully autonomous, achieved by combining optimised RNA chemistry, targeted delivery vehicles, and disease-specific payloads.

For conceptual clarity, three tiers of RNA system behaviour are distinguished throughout this Perspective. Programmable RNA systems are fixed at the design stage: sequence, chemistry, and delivery vehicle are configured before administration to produce a defined, pre-specified output, such as a chosen antigen or protein, and this describes the great majority of systems discussed here. Responsive RNA systems retain this fixed molecular design but their expression level, timing, or duration is modulated by the local delivery or disease environment encountered after administration, for example through tissue-selective LNP biodistribution or expression cassettes sensitive to local physiological cues. Self-adaptive RNA systems, in the fullest sense intended by the term, would sense a change in disease state after administration and autonomously adjust their molecular output without external redesign; no currently approved or clinically tested system meets this stricter definition. The term “self-adaptive” is therefore used in this Perspective in a forward-looking, aspirational sense to describe the trajectory of the field, while the concrete evidence reviewed below concerns programmable and, in some cases, responsive systems.

Self-amplifying RNA may extend antigen or protein expression at lower doses by encoding replicase machinery derived from RNA viruses (13), a mechanism no longer confined to preclinical models: the ARCT-154 self-amplifying RNA vaccine received the first regulatory approval for this platform class, and pooled phase 1–3 trials and subsequent phase 3 booster data have confirmed its safety and durable immunogenicity relative to conventional mRNA comparators (5, 6). Circular RNA may improve molecular stability and prolong expression duration, although clinical validation remains limited (14). Sequence-engineered mRNA enhances translation efficiency and reduces innate immune activation (4). Selective organ-targeting nanoparticles demonstrate that tissue biodistribution can be redirected by altering LNP composition (15). **Table 1** summarises the principal design features and translational challenges of these platforms, and **Table 2** maps each operational mode in **Figure 1** to a specific, published clinical or preclinical example, so that the conceptual framework is anchored throughout to concrete evidence rather than presented in the abstract. **Figure 1** illustrates how these platforms may be integrated within a programmable therapeutic framework.

**Table 1:** Potential design features of programmable RNA-based therapeutic platforms.

Platform feature	Main function	Possible application	Key translational challenge
Antigen-encoding mRNA	Immune priming	Infectious disease and cancer vaccines	Durability and immune escape
Self-amplifying RNA	Extended expression at lower RNA dose	Vaccines and protein expression	Reactogenicity and manufacturing complexity
Circular RNA	Improved molecular stability	Long-acting RNA therapeutics	Translation control and clinical validation
Sequence-engineered mRNA	Improved translation; reduced innate immune sensing	Protein replacement	Balancing expression and immunogenicity
Targeted lipid nanoparticles	Tissue-directed RNA delivery	Liver, spleen, lung, tumour, and immune-cell targeting	Extrahepatic delivery and species differences
Immunostimulatory formulation	Vaccine adjuvanticity	Cancer and infectious disease vaccines	Excess inflammation
Non-inflammatory formulation	Immune tolerance induction	Autoimmune disease	Durable tolerance induction
Personalised antigen-encoding mRNA combined with pembrolizumab	Vaccination combined with treatment	Cancer, infection, and inflammatory disease	Dose optimisation and regulatory classification

LNP, lipid nanoparticle; saRNA, self-amplifying RNA; CAR, chimeric antigen receptor.

**Table 2:** Illustrative clinical and preclinical examples mapped to the four operational modes in **Figure 1**.

Operational mode	Platform feature involved	Illustrative example	Evidence level	Source
Vaccine mode	Self-amplifying RNA	First regulatory approval of a self-amplifying RNA COVID-19 vaccine (ARCT-154); confirmed durable immunogenicity in pooled phase 1–3 and phase 3 booster trials	Approved	(5, 6)
Treatment mode	Personalised antigen-encoding mRNA combined with pembrolizumab, personalised antigen encoding	Individualised neoantigen mRNA therapeutic plus pembrolizumab reduced recurrence risk by 44% in resected high-risk melanoma (randomised phase 2b)	Clinical	(9)
Treatment mode	Personalised antigen-encoding mRNA	Personalised mRNA neoantigen vaccine in pancreatic cancer; T-cell responders showed longer recurrence-free survival than non-responders	Clinical	(10)
Tolerance mode	Non-inflammatory formulation	Non-inflammatory mRNA vaccine suppressed experimental autoimmune encephalomyelitis via antigen-specific tolerance	Preclinical	(16)
Reprogramming mode	Targeted LNP delivery, transient expression	LNP-delivered mRNA generated CAR T cells *in vivo* in a preclinical cardiac-injury model	Preclinical	(12)

This table is intended to be read alongside **Figure 1** and **Table 1**: each row anchors one operational mode of the conceptual framework to a specific, citable clinical or preclinical result. The Evidence level column indicates whether the cited example has achieved regulatory approval, has been tested in a registered clinical trial, or remains at the preclinical stage, since the examples span a substantial range of translational maturity.

**Figure 1:**
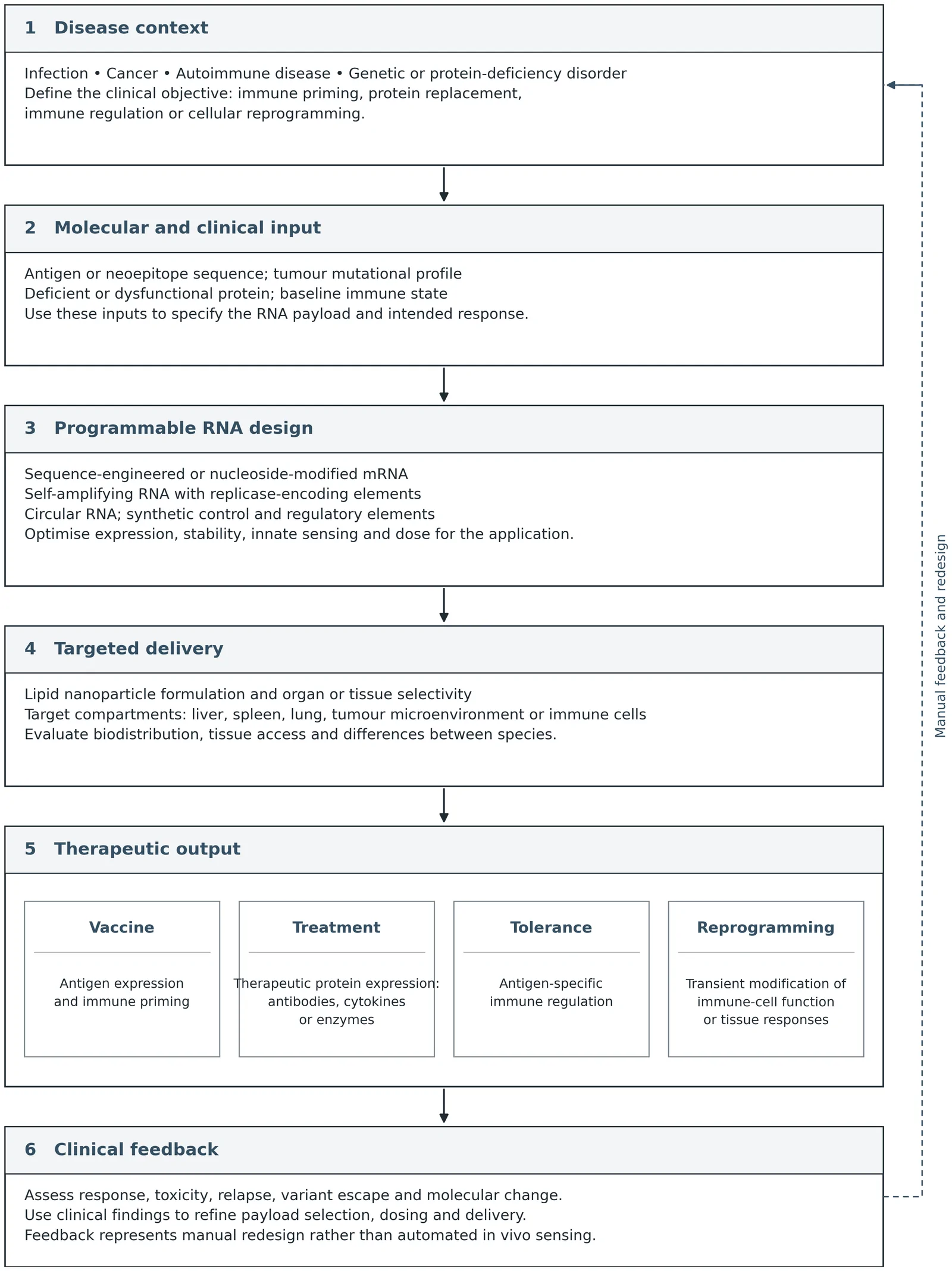
Conceptual framework for self-adaptive RNA-based therapeutics. The framework proceeds from disease context, through molecular and clinical input, programmable RNA design, and targeted delivery, to four operational therapeutic output modes (vaccine, treatment, tolerance, and reprogramming), each illustrated in this revision with a specific published clinical or preclinical example (see **Table 2**), and concludes with a clinical feedback stage. The dashed feedback arrow denotes an iterative redesign loop that is currently executed by investigators between dosing cycles rather than an autonomous biological process; this distinction is central to the manuscript’s argument that current systems are programmable before administration rather than self-adaptive after delivery. LNP, lipid nanoparticle; saRNA, self-amplifying RNA; CAR, chimeric antigen receptor.

## Therapeutic opportunities beyond infectious disease

Cancer is the most clinically advanced non-infectious application of mRNA therapeutics. Because tumours are genetically heterogeneous and evolve under therapeutic pressure, personalised mRNA vaccines encoding patient-specific neoantigens allow treatment to be matched to individual mutational profiles (7, 8). This matching strategy has now produced controlled clinical evidence of benefit rather than immunogenicity alone: in the KEYNOTE-942 phase 2b trial, an individualised neoantigen mRNA therapeutic combined with pembrolizumab reduced the risk of recurrence or death by 44% compared with pembrolizumab monotherapy in resected stage III/IV melanoma (9), and in pancreatic cancer, patients who mounted a measurable T-cell response to a personalised mRNA vaccine had longer recurrence-free survival than non-responders (10). Future platforms may combine neoantigen vaccination with short-term expression of immune-modulatory proteins to counteract tumour immune evasion, and with computational, AI-assisted neoantigen prioritisation to shorten the sequencing-to-dose timeline that currently constrains personalised manufacturing.

Autoimmune disease requires the opposite immunological objective. Rather than stimulating immunity, mRNA may be configured to induce tolerance. Krienke et al. (16) showed that a non-inflammatory mRNA vaccine suppressed experimental autoimmune encephalomyelitis by promoting antigen-specific immune tolerance, supporting the principle that mRNA can be engineered either to activate or to restrain immune responses.

Genetic and protein-deficiency disorders represent a further therapeutic opportunity. Because mRNA expression is transient, it may be preferable to permanent genome modification in settings where temporary protein replacement is clinically sufficient. However, repeat dosing, durability of expression, and tissue-specific delivery must be resolved before broad clinical application.

## Delivery as the central bottleneck

Delivery remains the primary limitation for programmable mRNA therapeutics. Although LNPs made mRNA vaccination clinically feasible, most current formulations preferentially distribute RNA to the liver or immune-rich tissues. Self-adaptive treatment will require more precise delivery to pulmonary, cardiac, tumour, lymph-node, and immune-cell compartments. Selective organ-targeting nanoparticles demonstrate that biodistribution can be altered by formulation design (15), yet endosomal escape efficiency, repeat-dosing tolerability, inflammatory potential, and human translatability remain incompletely resolved.

## Safety, regulation, and ethics

Products that combine immune priming, therapeutic protein expression, and targeted delivery may not fit neatly into existing regulatory categories for vaccines or biologics. This tension is no longer hypothetical: individualised neoantigen mRNA therapeutics such as mRNA-4157 sit between the vaccine and biologic categories, since they are manufactured per patient from tumour sequencing data yet regulated and reviewed largely within oncology biologic pathways rather than as vaccines (9). Safety assessment for self-adaptive mRNA therapeutics should therefore encompass RNA sequence design, encoded protein function, biodistribution, expression duration, innate immune activation, repeat-dosing effects, and off-target activity.

Equitable access warrants equal attention. Personalised mRNA medicines may depend on tumour sequencing, bioinformatics infrastructure, cold-chain logistics, and advanced manufacturing capacity; published cost estimates place current per-patient production costs for individualised neoantigen therapeutics in excess of $100,000, with industry analyses reporting fully personalised formulations reaching approximately $100,000-300,000 depending on sequencing, manufacturing, and logistics overheads (17), a figure that has direct implications for reimbursement policy and for feasibility in low- and middle-income settings. Without deliberate access strategies, including regional manufacturing hubs and tiered pricing models, these platforms risk widening global health inequalities.

## Research priorities

Future research should distinguish clearly between programmable design and genuine biological adaptivity. Delivery platforms should be evaluated in clinically relevant models and human tissues. Comparative studies are needed to determine when conventional mRNA, saRNA, or circular RNA is most appropriate, ideally through head-to-head trials rather than cross-trial comparison, since saRNA has already demonstrated non-inferior or superior immunogenicity to conventional mRNA at a fraction of the dose in phase 3 settings (6). In oncology, rational combinations with checkpoint inhibitors or adoptive cell therapies merit systematic evaluation, alongside biomarker-defined patient selection strategies informed by the differential outcomes already observed between immune responders and non-responders (10). In autoimmune disease, tolerogenic formulations should be prioritised. Regulatory science should develop clear classification frameworks for RNA products that function simultaneously as vaccines and therapeutics, and manufacturing science should prioritise cost reduction and regional production capacity to support equitable global deployment.

## Discussion

RNA technology has already transformed vaccinology, and its next phase is being defined by therapeutic convergence. The same platform that encodes protective antigens can also encode antibodies, cytokines, enzymes, or receptors, creating a flexible design space in which prevention and treatment become linked outputs of programmable RNA-based therapeutics. What has changed since earlier conceptual accounts of this convergence is the emergence of controlled clinical evidence at both ends of the spectrum: the first regulatory approval of a self-amplifying RNA vaccine confirms that programmable RNA chemistry can now clear the full path from design to licensure (5), while a randomised phase 2b reduction in melanoma recurrence risk with an individualised neoantigen mRNA therapeutic (9) shows that the treatment end of the spectrum is likewise moving beyond immunogenicity signals towards demonstrable clinical benefit.

This clinical progress sharpens, rather than resolves, three practical questions. First, regulatory classification: individualised, per-patient mRNA therapeutics do not map cleanly onto either vaccine or conventional biologic review pathways, and the absence of a bespoke framework risks inconsistent evidentiary requirements across agencies and slower patient access. Second, patient selection: the observation that immune responders to personalised pancreatic cancer vaccines have better recurrence-free survival than non-responders (10) argues for early biomarker-defined trial designs rather than unselected cohorts, so that resources are directed towards patients most likely to benefit. Third, manufacturing economics: per-patient production costs reported in the low hundreds of thousands of US dollars for individualised neoantigen therapeutics are compatible with high-income adjuvant oncology settings but are not compatible, without substantial cost reduction or technology transfer, with deployment in low- and middle-income countries, where much of the global cancer and infectious disease burden falls.

Premature claims should still be avoided. Most current mRNA systems remain programmable before delivery rather than autonomous within the body; the clinical feedback loop depicted in **Figure 1** is at present executed by investigators between dosing cycles, informed by sequencing, imaging, and immune-monitoring data, rather than by any biological sensing mechanism intrinsic to the RNA itself. A realistic near-term pathway therefore remains modular adaptivity: combining disease-specific RNA payloads, targeted nanoparticles, and controllable expression profiles, now increasingly supported by human outcome data rather than preclinical rationale alone. Over time, synthetic RNA control elements, coupled with computational neoantigen and biomarker prediction, may support more responsive therapeutic systems. The framework proposed here does not supplant existing vaccines or biologics but extends their capabilities towards responsive, personalised, and temporally controlled therapy, and its priorities for the next several years are regulatory clarification, biomarker-guided trial design, and manufacturing cost reduction to enable equitable access.

## Conclusion

Programmable mRNA therapeutics represent a credible next frontier in RNA-based nanomedicine, and this claim now rests on more than conceptual extrapolation. By integrating vaccination, transient protein expression, immune modulation, and precision delivery, mRNA platforms have already advanced from static prophylaxis towards responsive disease treatment validated in controlled human trials at both the prophylactic and oncology-therapeutic ends of the spectrum, as detailed above. This progress validates the convergence thesis empirically rather than only theoretically, while confirming that genuine biological self-adaptivity, in the sense of RNA systems that sense and respond within the body without external redesign, remains an aspirational rather than a current capability. Realising the fuller potential of this field will require continued delivery precision, rigorous safety validation, biomarker-guided patient selection, RNA-specific regulatory classification, and a sustained, resourced commitment to equitable manufacturing and access, particularly for the low- and middle-income settings that stand to gain the most from responsive, lower-cost RNA-based nanomedicine.

## Data Availability

The original contributions presented in the study are included in the article/supplementary material. Further inquiries can be directed to the corresponding author.
